# Cost-Effectiveness of Introducing Nuvaxovid to COVID-19 Vaccination in the United Kingdom: A Dynamic Transmission Model

**DOI:** 10.3390/vaccines13020187

**Published:** 2025-02-14

**Authors:** Clive Pritchard, Lucie Kutikova, Richard Pitman, Kira Zhi Hua Lai, Hadi Beyhaghi, IIana Gibbons, Amanda Erbe, Marija Živković-Gojović, Catherine Cosgrove, Mark Sculpher, David Salisbury

**Affiliations:** 1 ICON Clinical Research, Reading RG2 6AD, UK; 2 Novavax, 8001 Zürich, Switzerland; 3 ICON Clinical Research, Toronto, ON L7N 3G2, Canada; 4 Novavax, Gaithersburg, MD 20878, USA; 5 Novavax, Reading RG2 6GP, UK; 6 RTI Health Solutions, Research Triangle Park, NC 27709, USA; 7 St. George’s University Hospital, London SW17 0QT, UK; 8 Centre for Health Economics, University of York, York YO10 5DD, UK; 9 Royal Institute of International Affairs, Chatham House, London SW1Y 4LE, UK

**Keywords:** SARS-CoV-2, COVID-19 vaccines, dynamic transmission, cost-effectiveness, cost-utility, COVID-19, economics

## Abstract

**Background/Objectives:** Vaccination against SARS-CoV-2 remains a key measure to control COVID-19. Nuvaxovid, a recombinant Matrix-M–adjuvanted protein-based vaccine, showed similar efficacy to mRNA vaccines in clinical trials and real-world studies, with lower rates of reactogenicity. **Methods:** To support decision making on UK vaccine selection, a population-based compartmental dynamic transmission model with a cost-utility component was developed to evaluate the cost-effectiveness of Nuvaxovid compared with mRNA vaccines from a UK National Health Service perspective. The model was calibrated to official epidemiology statistics for mortality, incidence, and hospitalisation. Scenario and sensitivity analyses were conducted. **Results:** In the probabilistic base case, a Nuvaxovid-only strategy provided total incremental cost savings of GBP 1,338,323 and 1558 additional quality-adjusted life years (QALYs) compared with an mRNA-only vaccination strategy. Cost savings were driven by reduced cold chain-related operational costs and vaccine wastage, while QALY gains were driven by potential differences in vaccine tolerability. Probabilistic sensitivity analysis indicated an approximately 70% probability of cost-effectiveness with Nuvaxovid-only versus mRNA-only vaccination across most cost-effectiveness thresholds (up to GBP 300,000/QALY gained). **Conclusions:** Nuvaxovid remained dominant over mRNA vaccines in scenario analyses assessing vaccine efficacy waning, Nuvaxovid market shares, and the vaccinated population.

## 1. Introduction

As of December 2023, approximately 24 million people had been infected by SARS-CoV-2 in the UK, leading to over 200,000 premature deaths [1]. Older individuals and those with underlying comorbidities are among those at disproportionate risk of severe COVID-19, hospitalisation, and death, following SARS-CoV-2 infection [2,3].

Although vaccination programmes have substantially reduced the risk of SARS-CoV-2 infection and COVID-19–related death, there remains a substantial economic and healthcare burden associated with COVID-19, with over 170,000 hospitalisations reported in England by the UK Health Securities Agency (UKHSA) in 2023 [3].

These figures highlight the ongoing need to maintain effective control of COVID-19. Recommendations for vaccination from the UK Joint Committee for Vaccination and Immunisation (JCVI) for the Autumn 2024 vaccination programme included vaccination for individuals aged ≥65 years, and those aged 6 months to 64 years and in a clinical risk group according to the COVID-19 chapter of the Green Book [4,5]. Vaccine options in the Autumn 2024 programme included two mRNA vaccines, Comirnaty^®^ (Pfizer-BioNTech, Cambridge, MA, USA) and Spikevax^®^ (Moderna Inc., Cambridge, MA, USA), administered by the UKHSA [4,6].

Since the pandemic’s onset, several SARS-CoV-2 variants have emerged, resulting in potential changes in COVID-19 severity and vaccine efficacy [7,8,9]. Vaccines therefore need to be adapted to ensure immunogenicity and effectiveness against currently circulating variants. Due to the unpredictability of new SARS-CoV-2 variants, and as adapted vaccines gain regulatory approval by the UK Medicines and Healthcare products Regulatory Agency (MHRA), it will be important to ensure that vaccination programmes remain diverse and comprehensive to ensure resilience against future outbreaks.

Nuvaxovid™ (Novavax, Inc., Gaithersburg, MD, USA) is a recombinant protein vaccine containing the SARS-CoV-2 spike protein, and Matrix-M™ (Novavax, Inc., Gaithersburg, MD, USA), a saponin-based adjuvant [10]. Nuvaxovid, including variant-adapted versions, has been authorised for use by regulatory bodies in 40 countries, including the UK, to prevent COVID-19 in individuals aged 12 years and over [11]. Both the original and variant-adapted Nuvaxovid showed similar efficacy to mRNA COVID-19 vaccines in clinical trials with an acceptable tolerability profile [12,13,14,15], while equivalent effectiveness to Comirnaty was demonstrated in real-world cohort studies [16,17]. Additionally, Nuvaxovid can be stored in a standard refrigerator at 2 °C to 8 °C (i.e., is refrigerator stable) for up to 12 months [11]. By contrast, mRNA vaccines require a cold or ultracold supply chain (−15 °C for Spikevax; −60 °C for Comirnaty) [14,18].

These differentiating factors suggest that there may be advantages to using Nuvaxovid in the UK vaccination programme. Data on the health and economic impact of COVID-19 and related interventions can help to inform decision making to decrease the ongoing health burden of COVID-19 [19]. While other health economics analyses assessed cost-effectiveness of COVID-19 vaccinations relative to no vaccination [20,21,22], comparisons among vaccines are limited.

This study aimed to assess the cost-effectiveness of introducing protein-based vaccination with Nuvaxovid to the annual UK COVID-19 vaccination strategy, using a dynamic transmission approach to capture indirect vaccination effects. This study provides estimates of the potential impact on healthcare costs and quality-adjusted life years (QALYs) gained with use of a Nuvaxovid-only compared with an mRNA-only vaccination strategy. The population considered was as per the Nuvaxovid indication and within the JCVI Autumn 2024 recommendations: those aged ≥65 years and those aged 12–64 years with pre-existing health conditions that would increase the risk of severe COVID-19 outcomes [4,11].

## 2. Methods

**Figure 1:**
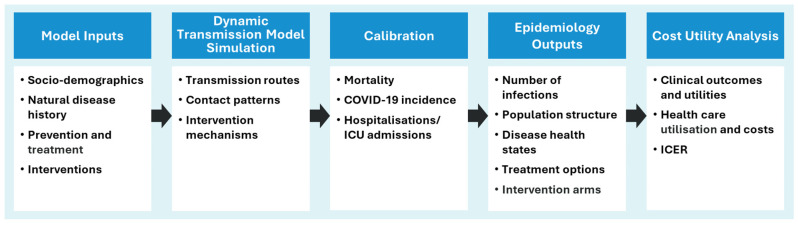
Schematic representation of the model concept. ICER = incremental cost-effectiveness ratio; ICU = intensive care unit.

To comprehensively evaluate the cost-effectiveness of Nuvaxovid compared with mRNA vaccines for the UK vaccination programme, a model was developed consisting of: (1) An epidemiological component, using dynamic transmission to simulate the transmission of SARS-CoV-2 in the UK, and (2) a health economics component, to provide a cost-utility analysis. The model concept is represented in Figure 1.

The dynamic transmission model simulates SARS-CoV-2/COVID-19 disease dynamics, producing estimates of the number of vaccines administered, the number of asymptomatic and symptomatic infections, and the number of COVID-19–related deaths. Upon exiting the dynamic transmission model, the population with symptomatic infection are assigned to disease health states including hospitalisation (non-intensive care unit [ICU] and ICU admissions) and long COVID status, based on probabilities determined from official statistics or the published literature. The model was calibrated for mortality, incidence and hospitalisation, including ICU admissions, prior to the cost-utility analysis. Because the calibration steps were performed using population-based data inputs, the dynamic transmission simulation reflects the UK population including individuals with existing immunity due to either prior SARS-CoV-2 infection and/or previous vaccination (individuals would have received a mixture of mRNA and vector-based vaccines). Then, in the cost-utility (economic) component of the model, healthcare resource use (HCRU) and other healthcare-related costs are applied, as well as the impact of COVID-19, including hospitalisation and ICU admission, on quality of life, expressed on the disutility scale. Vaccine-related costs and disutility associated with tolerability events are also applied in the cost-utility analysis, based on the “number of vaccines administered” output of the dynamic transmission model simulation.

The model compares a vaccination strategy with Nuvaxovid only to vaccination with mRNA vaccines only. mRNA vaccines were selected as the comparator because they are the most administered COVID-19 vaccines in the UK and the only vaccines used by the National Health Service (NHS) [4,6]. In the base case, 100% market share of Nuvaxovid was assumed, and scenario analyses investigated various market shares for Nuvaxovid and mRNA vaccines. Analysis was performed from the perspective of the NHS, with a cost-effectiveness threshold of GBP 20,000/QALY. Probabilistic sensitivity analysis (PSA) with 10,000 iterations was used for the base case and scenario analyses.

The model time horizon was 1 year, and vaccines were assumed to be administered once, at the beginning of the modelled 12-month period. The single administration was selected due to the potential variability in the timing of vaccine administration among different populations—the JCVI recommends that the vaccine should usually be offered no earlier than around 6 months after the previous vaccine dose, although this can be performed earlier, especially for populations at very high risk of severe outcomes such as immunocompromised individuals [4]. Because of the potential variability in the timing of vaccine administration among different populations, we opted to model the annual vaccination in the autumn. The aim was to capture the impact of vaccination on winter pressures when it would have the greatest effect.

The model’s design and data inputs were informed by 53 studies identified in a targeted literature review. Searches were performed in Embase and Medline to identify relevant dynamic transmission studies and cost-effectiveness analyses of vaccination against COVID-19 and other respiratory viruses, such as influenza or respiratory syncytial virus. Searches included clinical (efficacy, waning, tolerability) and utility inputs, healthcare resource use and costs (storage costs). A pragmatic approach was adopted, in which the most recent publications relevant to the UK were selected (41/600 studies published from 2019 onwards). Data gaps were then addressed using studies from other countries (12 studies). To ensure the most recent data were used to populate the model, the targeted literature review was supplemented with literature searches for utility inputs (8 studies), official statistics relating to the pandemic (e.g., COVID-19 deaths), and standard reference sources for costs (e.g., NHS Reference Costs, Personal Social Services Research Unit [PSSRU] Unit Costs). Criteria applied to select the best matching comparable data sources for Nuvaxovid and mRNA vaccines were study design, follow-up, endpoint definitions, and period of data collection.

### 2.1. A Dynamic Transmission Model

**Figure 2:**
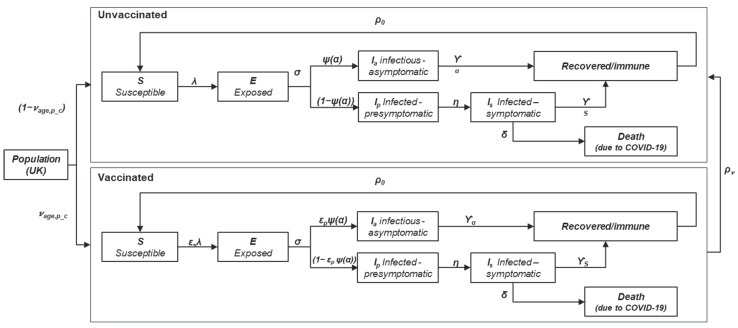
Structure of the dynamic transmission model.

A population-based dynamic transmission approach was selected to capture the indirect effects of vaccination in the overall UK population, in accordance with best practice recommendations from ISPOR (2018) and guidance from ISPOR-SMDM (2012) [23,24]. Dynamic transmission methodology is recommended because vaccination programmes impact the force of infection (the susceptibility to infection of vaccinated individuals), resulting in herd immunity and changing the disease dynamics in the wider population [23]. As static models do not capture these indirect herd immunity effects of vaccination, they may not accurately capture the full epidemiological and economic impact of an intervention [23,24]. The dynamic transmission approach accounts for contact patterns across age groups, which were obtained from the Great Britain Close Contact matrix based on the POLYMOD study (Supplementary Figure S1) [25,26]. The structure of the dynamic transmission model was based upon methodologies from previously published COVID-19 models [27,28] and was adapted to include a variety of vaccine profiles and combinations of vaccination strategies (Figure 2).

In the model, the population is divided into compartments representing health/disease states. Initially, the population is categorised as either susceptible to the infection, or having residual immunity due to either prior exposure to the virus or reception of a vaccine dose(s) in the previous year. Vaccines are administered prior to initiation of the disease dynamics component of the model. The cumulative COVID-19 vaccination rate was obtained from UKHSA weekly national influenza and COVID-19 surveillance reports (Table 1). Uptake of COVID-19 vaccine ranged from 5.34% in those aged 12–18 years with pre-conditions, to 75.7% in those aged ≥80 years [29,30,31].

The dynamic transmission model includes five disease-related states, with the probability of progressing through each state depending on age and vaccination status (Figure 2):

- **Susceptible (*S*):** The population in this compartment are susceptible to infection, i.e., not immune. Susceptibility to infection is impacted by vaccination status. Transmission probability (chance of becoming infected) is dependent on age and contact patterns.
- **Exposed (*E*):** The condition of being infected, but not yet infectious (the latent period).
- **Infectious (*I*):** The population in the ***E*** state moves to either the infectious asymptomatic state (***I_a_***) or the infectious presymptomatic state (***I_p_***). All individuals in ***I_p_*** will progress to the infectious symptomatic state (***I_s_***). The proportion of asymptomatic infections is dependent on age group and vaccination status.
- **Recovered/immune:** The population compartments with either asymptomatic or symptomatic infection progresses to the recovered/immune state, and subsequently returns to the ***S*** state according to the rate of immunity waning, diminishing in an exponential manner from 100% to 31% at 12 months [27].
- **COVID-19–related death**: The population in the infectious symptomatic state may progress to death without recovering from COVID-19. The rate of COVID-19–related mortality is age dependent.

The resulting model is encapsulated within a system of ordinary differential equations. One key equation to highlight is that for the density-dependent force of infection—the instantaneous probability of infection per susceptible case. This equation consists of several elements including age-dependent contact frequencies $(c_{a,a^{\prime }}$); the transmission probability per established contact ($\xi_{a})$; susceptibility to infection according to vaccination status ($\varepsilon_{S}^{v})$; and proportionate reduction in virus transmissibility ($\varepsilon_{A}$) in asymptomatically or pre-symptomatically infected individuals, relative to symptomatically infectious cases and independent of vaccination status. The force of infection specific to the age group and vaccination status ($\lambda_{a,\nu }$) is described by the following equation:

$$\lambda_{a,\nu }=\xi_{a}\varepsilon_{S}^{v}\underset{a^{\prime }}{\sum }c_{a,a^{\prime }}\sum_{v=0}^{1}\frac{\left[\varepsilon_{A}(A_{t,a^{\prime }}^{v}+P_{t,a^{\prime }}^{v}\right)+I_{t,a^{\prime }}^{v}]}{N_{i}\left(t\right)}$$

Other equations included in the model can be found in Supplementary Figure S2. Key input parameters for the dynamic transmission model are included in Table 1.

### 2.2. Model Calibration

The mRNA-only arm of the model was calibrated using a two-step gradient descent methodology. During the calibration process, model parameters were adjusted iteratively in three main stages to align with the reported official statistics: Firstly, calibration was performed for COVID-19 deaths; secondly for incidence; and finally for the reported number of hospitalisations, including ICU admissions. Due to the lack of testing and thus risk of underreporting after 2022, 2022 data for the incidence and mortality was used. As testing in hospitals continued after this period, the most recently available data (April 2023–April 2024) were used to calibrate hospitalisations/ICU admissions. Following the full calibration process, the model outputs achieved proximity to the reported mortality, incidence and hospitalisation data (Supplementary Table S1).The initial demographic inputs were based on the UK population in 2021, with data on the population size for England from the ONS extrapolated to the full UK population [32]. Mortality and incidence were calibrated against data from 2022—the most recent period with widespread testing for COVID-19 before the testing requirements were lifted in early 2023 (Table 1). Since most COVID-19 reporting ceased in Wales, Scotland and Northern Ireland in mid-2022, COVID-19 incidence and deaths in England were extrapolated to all four nations of the UK using the relative ratio between the England and UK populations [34]. The COVID-19 incidence and mortality estimates for England in 2022 were 8.6 million COVID-19 cases and 33,494 deaths [35], including testing conducted by the NHS, UKHSA, government testing, antibody testing, and surveillance testing. Because testing in hospital was thought to be largely unaffected by the change in reporting requirements, hospitalisations and ICU admissions were based on the most recent 12-month period for which data were available at the time of the analysis (April 2023–April 2024). Reported data on hospital admissions were available for all four nations of the UK for most of the period; where data gaps existed, hospitalisation numbers were extrapolated from England to the UK (Table 1).

Once the initial calibration for mortality (number of COVID-19–related deaths) had been completed, with the model generating the expected number of deaths for England and adjusted to the full UK population, the number of incident cases was calibrated. As the relationship between incidence and mortality in the model was based on infection fatality rates estimated in a 2020 article by Ghisolfi et al. [36], once the mortality outputs had been calibrated, the modelled number of incident cases was expected to be consistent with the reported incidence data. Having confirmed that this was the case for England, with modelled estimates a close approximation of the observed incidence (8,651,622 modelled versus 8,651,710 reported), we proceeded to calibrate the model to generate the expected number of cases for the UK, taking account of the difference in population between England and the UK.

The final calibration step was to calibrate hospitalisation, including ICU admissions, to the April 2023–April 2024 statistics. UKHSA surveillance data on the relative numbers of hospital and ICU/high dependency (HDU) admissions per 100,000 population by age group were then used to represent the probability of ICU admission, given hospitalisation, with the model reporting the estimated numbers of non-ICU and ICU admissions. To match the time for the hospitalisation data as closely as possible, average admission rates were taken over the 52-week period up to 25 April 2024.

### 2.3. Cost-Utility Analysis

The economic analysis used a cost-utility approach, assigning HCRU, costs and utilities to the modelled population according to COVID-19 status (Figure 1). Considerations included vaccination strategies and clinical and cost inputs. The most recent COVID-19 vaccine uptake data for each population was used [29,30,31]. Based on clinical trials, parity was assumed for clinical efficacy parameters between Nuvaxovid and mRNA vaccines [13,15,37,38]. Tolerability was based on a meta-analysis of clinical trials, which suggested a more favourable rate of tolerability events for Nuvaxovid than mRNA vaccines; the rate of tolerability events was translated into a QALY gain in the model [39]. Freeze-related thawing, handling and monitoring costs, and cold chain further differentiate between Nuvaxovid and mRNA vaccines, in favour of Nuvaxovid, and were accounted for in the model.

#### 2.3.1. Vaccination Strategies

Individuals aged ≥65 years and those aged 12–64 years with pre-conditions were assumed to be eligible for vaccination, in line with the Nuvaxovid-approved indication [11] and within the population recommended by the JCVI in their Autumn 2024 recommendations [4]. Scenario analyses examined variations in the population age cut-off for vaccine eligibility and tested an exclusively risk-based strategy in which only individuals with pre-conditions aged 12–64 years received vaccination.

The base case compared vaccination with Nuvaxovid (100% market share) with mRNA vaccination (100% market share divided equally between mRNA vaccines). To address the policy question of most relevance to the JCVI, scenarios were also tested in which the Nuvaxovid market share was set to 10%, 33.3% and 50%, with the remaining market share divided equally between the two mRNA vaccines.

#### 2.3.2. Clinical and Utility Inputs

Clinical parameters and utility inputs including vaccine efficacy, efficacy waning, and tolerability, were identified based on a targeted literature review, and are summarised in Table 2. Criteria applied to select best matching comparable data sources for Nuvaxovid and mRNA vaccines were study design, follow-up, endpoint definitions, and period of data collection.

Clinical effectiveness parameters and vaccine efficacy waning were assumed to be equivalent between mRNA vaccines and Nuvaxovid in the base case, with reference to clinical trial data (Table 3) [13,38]. COVID-19 vaccines have been shown to reduce the probability of both asymptomatic and symptomatic infections with SARS-CoV-2 and the probability of severe disease [13,15,16,17,37,38]. To estimate a starting point for vaccine efficacy against asymptomatic and symptomatic infections, we used data for Spikevax from El Sahly et al. [38] and applied that estimate to mRNA vaccines and Nuvaxovid. El Sahly et al. [38], in a 2021 phase 3, randomised, placebo-controlled trial, estimated that the efficacy of Spikevax against asymptomatic and symptomatic SARS-CoV-2 infections was 82.0% (95% confidence interval (CI), 79.5%, 84.2%) [38]. The vaccine efficacy for Nuvaxovid against asymptomatic and symptomatic infections was similar, at 82.5% (95% CI, 75.0%, 87.7%), in the pivotal, randomised phase III study [13,15]. Equivalent vaccine effectiveness between Nuvaxovid and Comirnaty was also reported in a population-based comparative effectiveness study [16,17,37]. These clinical trials were chosen over more recent real-world evidence due to similarities in trial design (the trials were conducted for regulatory purposes), endpoint definitions (efficacy against asymptomatic and symptomatic infection), and the timing of data collection (July–October 2020 for Spikevax, and September–November 2020 for Nuvaxovid). Inclusion and formal synthesis of other studies was not possible due to differences in the time points at which the studies were conducted, with circulating SARS-CoV-2 variants evolving over time, and changing immunity in the general population due to vaccination or SARS-CoV-2 infection.

Although the level of starting vaccine efficacy may vary today compared with 2020, when El Sahly et al. and other primary vaccine clinical trials were conducted, the immunogenicity data from recent clinical trials of the variant-adapted COVID-19 vaccines demonstrate similar or improved levels of neutralising antibody titres to those observed during the selected trials [12]. For example, XBB.1.5-adapted Nuvaxovid achieved superior levels of antibody titres to the original vaccine [40]. Further, these assumptions do not impact the results and conclusions from the analysis because the same value was applied across Nuvaxovid and both mRNA vaccines. We tested potential uncertainty around vaccine efficacy in our sensitivity analyses, using the CIs from each individual trial.

Vaccine efficacy waning includes two parameters—the start/onset of waning, and the rate of waning. For the start of vaccine efficacy waning, the base case was set at 1 month based on data from a meta-analysis of real-world estimates of mRNA vaccines against Omicron SARS-CoV-2 variants [41]. The meta-analysis showed that vaccine efficacy against asymptomatic and symptomatic infections begins to decline in the first month after receiving a booster vaccination or after completing the primary vaccine series [41]. The same base case was selected for Nuvaxovid. However, clinical trials and real-world studies indicate that this may be a conservative estimate [12,13,42,43,44,45]; onset of efficacy waning for Nuvaxovid was therefore explored further in a scenario analysis. The rate of efficacy waning was set as exponential, calculated based on a pooled estimate of vaccine efficacy against all infections for the Omicron variant at 1, 3, 6, and 9 months after any booster dose of mRNA vaccines (Supplementary Figure S3) [41].

Based on real-world evidence from the European Centre for Disease Prevention and Control (ECDC), vaccine efficacy against severe disease was set at 50% [46]. Although clinical trials examined vaccine efficacy against severe COVID-19, the estimates are limited by the short follow-up period, resulting in a high efficacy against hospitalisation. Consequently, based on the real-world evidence estimate from the ECDC [46], we made a conservative assumption that the vaccine effectiveness against severe disease was 50% and was consistent for both Nuvaxovid and mRNA vaccines. The same proportion was applied to the reduction in COVID-19–related death. We tested potential uncertainty in these efficacy parameters in our sensitivity analyses.

Utility decrements for symptomatic COVID-19 and associated events were assumed to be represented by those reported for influenza [47], whereas baseline utilities (without COVID-19) were based on the EQ-5D population norms for England [48] (Table 2). As differences have been reported between the reactogenicity/tolerability profiles of the vaccines [39,49,50,51], fewer QALY losses were anticipated following vaccination with Nuvaxovid versus mRNA vaccines. Rates of tolerability events were taken from a meta-analysis of COVID-19 vaccine RCTs, including mRNA, protein-based, and other vaccine types [39]. The reported numbers of adverse vaccine effects per vaccinated person were 2.497 and 1.572 for mRNA vaccines and Nuvaxovid, respectively (Table 2 and Supplementary Table S2) [39]. Using rates from the meta-analysis combined with a utility loss of 0.05 per day (for 1 day), based on influenza vaccine-related adverse events in a 2016 article by Leung et al. [52], we estimated that each protein vaccination would be associated with a QALY loss of 0.00022, compared with 0.00034 for mRNA vaccination (Table 2).

The disutility assumptions are consistent with results from other studies [49,51], and real-world evidence [50], which were aligned with the results of the meta-analysis [39]. Specifically, a real-world study showed that, on average, Nuvaxovid recipients reported 1.8 systemic reactogenicity symptoms, whereas the corresponding number for mRNA vaccine recipients was 3.2 symptoms [50]. Similarly, the NIAID and COV BOOST trials suggested that mRNA vaccines may be associated with higher rates of local and systemic tolerability events compared with protein-based Nuvaxovid [49,53,54,55]. We followed a conservative approach, only including the rates of tolerability events in QALY calculations; the potential advantage in tolerability on resource use and costs was not included.

**Table 2:** Base case clinical and utility inputs for the cost-utility component of the model.

Parameter	Base Case Value
**Vaccine efficacy, waning and tolerability parameters**	
Initial vaccine efficacy against all infections [38]	82%
Start of waning post vaccination [41]	1 month
Vaccine efficacy waning per month [41]	0.08
Efficacy against severe disease [46]	50%
Nuvaxovid average adverse events/recipient	1.572
mRNAs average adverse events/recipient	2.497
**Utility decrements**	
Symptomatic case [47]	0.00800
Non-fatal hospitalisation [56]	0.02010
Non-fatal ICU [27]	0.15
Long COVID [57]	0.13
Nuvaxovid tolerability [39,52] *	0.00022
mRNA tolerability [39,52] *	0.00034

ICU = intensive care unit; * vaccine-related adverse events were assumed to last 1 day.

#### 2.3.3. Healthcare Resource Use and Cost Inputs

Healthcare resource use and cost inputs were identified via the targeted literature review, as summarised in Table 3. Costs of vaccine acquisition and administration were based on official list pricing; pricing was assumed to be the equal for mRNA vaccines and Nuvaxovid, although this may not reflect the true final costs after confidential discounts [58,59]. However, Nuvaxovid is refrigerator stable for up to 12 months [11], whereas mRNA vaccines require storage in the freezer (−60 °C [Comirnaty [60]] or −15 °C [Spikevax [18]]). Hence, both mRNA vaccines need to be thawed and monitored for their shelf-life expiry after thawing (30 days for Spikevax and 10 weeks for Comirnaty) [11,14,18]. The additional resource use for these activities has been accounted for in the model (Table 3). Additionally, depending on the cold chain and temperature required, there may be wastage during transportation, with more wastage occurring with lower temperature requirements. In our analysis, we used resource use and wastage associated with vaccine cold storage requirements based on an average of the costs reported for mRNA vaccines; for Nuvaxovid, costs reported for the Janssen COVID-19 vaccine, which has similar storage requirements, were used [61]. Potential variability in these costs was accounted for in sensitivity analyses.

NHS HCRU costs associated with COVID-19 include hospitalisation costs, with and without the use of the ICU, outpatient visits, accident and emergency (A&E) visits, and primary care costs. In the cost-utility analysis, these costs were applied per case of symptomatic COVID-19, or to a proportion of symptomatic cases: 15.5% of cases were assumed to incur the cost of a GP visit and 2.7% an A&E visit, both based on Sandmann et al. (2022) [62] (Table 3). We opted to use these data as they were collected during the period when COVID-19 testing was being systematically conducted. We addressed potential variability in these proportions in the sensitivity analyses. As COVID-19 testing is still being routinely conducted in hospitals, we used the most recent hospitalisation and ICU data available at the time of the analysis, up to April 2024 [63].

The impact and definition of long COVID are constantly evolving based on emerging studies examining the long-term impact of COVID-19. We selected data used by the External Assessment Group (EAG) from the National Institute for Health and Care Excellence (NICE) report in August 2023, which assumed that 10% of the high-risk COVID-19 patients who were not hospitalised experience long COVID after Omicron infection, requiring follow-up healthcare [57] (Table 3).

The cost associated with a GP visit was derived from PSSRU unit costs, with hospital outpatient and inpatient care costs being sourced from NHS Reference Costs (Table 3). ICU costs are based on an estimate of the cost per bed-day from NHS Reference Costs combined with a mean ICU stay of 10 days [64,65]. Since healthcare costs were reported in 2022, prices were uprated by 1.078 to 2023 prices using the consumer price inflation rates for the health division as reported by the Office for National Statistics [66].

**Table 3:** Cost and healthcare resource use inputs.

Parameter	Base Case Value		
	Proportion		Cost per Case
**Vaccine-Related Costs**			
Cost of vaccines [58]	—		GBP 71.00
Vaccine administration costs [59]	—		GBP 7.54
Cold chain transportation wastage [61]	Nuvaxovid: 0.02%mRNA: 0.10%		—
Freeze-related costs (thawing, handling, monitoring of thawed vials) * [67,68]	—		Nuvaxovid: GBP 0 mRNA: GBP 0.14
**Healthcare resource use costs**			
General practitioner visit [62,69]	15.5%		GBP 44.20
Accident and emergency (emergency department) visits [62,65]	2.7%		GBP 260.88
Hospitalisation by age [65,70,71]	0.09–11.89%		GBP 3533.68
ICU hospitalisation by age [63,64,65,70]	5.54–0.58%		GBP 24,494.10 ($2449.41/day, 10 days)
Post-hospitalisation (ICU and non-ICU) care [57]	—		GBP 413.95
Long COVID care [57]	10%		GBP 2515.46

ICU = intensive care unit; reported costs were adjusted by the consumer price index to 2023 GBP; * time spent multiplied by the average salary of the nurse and pharmacist.

### 2.4. Scenario and Sensitivity Analyses

Scenario analyses included variation in the rate of waning of efficacy for Nuvaxovid; setting the Nuvaxovid market share to 10%, 33.3% and 50%; and the population eligible for annual vaccination, to account for any future changes in JCVI recommendations.

The base case assumed equal efficacy waning for each vaccine, including when waning starts, and rate of waning. Scenario analysis started waning for Nuvaxovid at 2 months after vaccination (compared with 1 month for mRNA vaccines). This was based on data from a retrospective cohort study in Italy in which the effectiveness of Nuvaxovid against asymptomatic and symptomatic COVID-19 infections remained stable for 2 months [72]. This assumption is further supported by other studies of Nuvaxovid primary series or booster in which durable protection was demonstrated for 3–11 months [12,42,43,44,45,73].

Probabilistic sensitivity analysis (PSA) was conducted with 10,000 simulations of mean costs and QALYs to ensure consistency in reporting, even though some scenarios reached convergence before the final iteration. Deterministic sensitivity analysis (DSA) investigated the impact of varying input parameter values one parameter at a time between lower and upper bounds relative to the base case value. Parameters varied in the PSA and DSA are included in Supplementary Tables S3 and S4.

## 3. Results

### 3.1. Probabilistic Results

In the base case, using probabilistic sensitivity analysis, vaccination with Nuvaxovid was estimated to generate total incremental cost savings of GBP 1,338,323 (95% credible interval: −GBP 225,070,810, GBP 221,780,060) and an additional 1558 QALYs (95% credible interval: −38,215 QALYs, 41,067 QALYs), resulting in dominance over the mRNA-only vaccine strategy (Table 4). The QALY gains were due to the potential lower rates of tolerability events with Nuvaxovid versus the mRNA vaccines; rates of tolerability events were based on a meta-analysis of the pivotal trials [39]. The lower costs resulted from differences in storage temperature and cold chain requirements between Nuvaxovid and mRNA vaccines—as Nuvaxovid is refrigerator stable, costs associated with thawing, handling and monitoring of thawed vials are avoided.

### 3.2. Sensitivity Analyses

**Figure 3:**
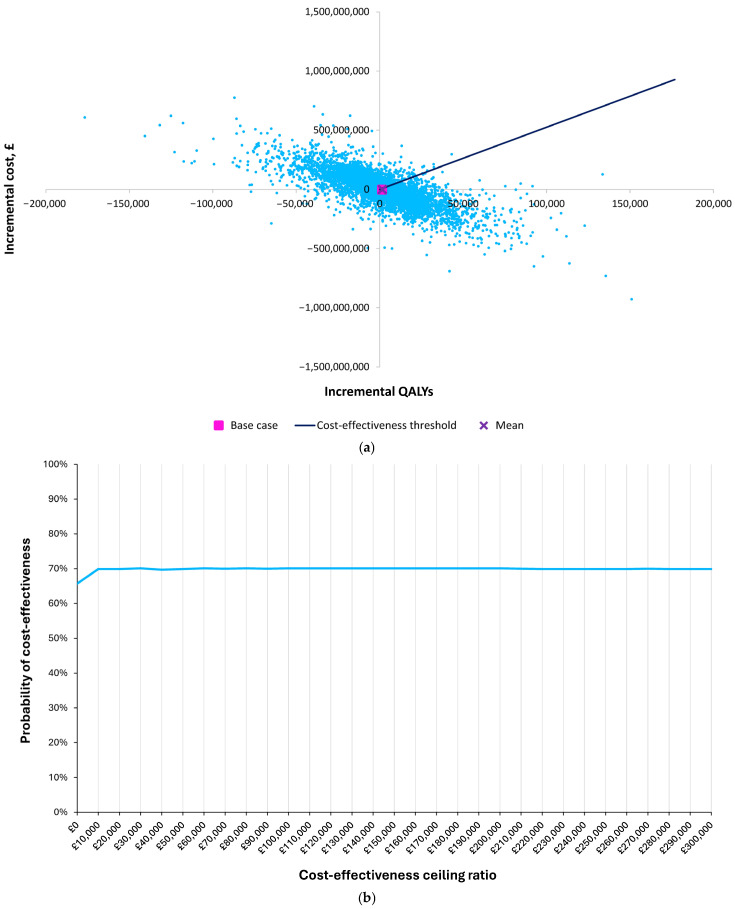
(**a**) Results of the probabilistic sensitivity analysis under a cost-effectiveness threshold of GBP 20,000; (**b**) cost-effectiveness acceptability curve.

The Nuvaxovid-only strategy had a 70% probability of being cost effective at a GBP 20,000/QALY threshold. The estimated total costs and QALYs associated with using Nuvaxovid compared with mRNA vaccines for annual COVID-19 vaccination resulted in 70% of 10,000 simulations of the incremental cost-effectiveness ratio (ICER) falling under the GBP 20,000/QALY cost-effectiveness threshold (Figure 3a). The key proportion is that relating to simulations below, i.e., ‘Southeast’, of the line representing the cost-effectiveness threshold, which is set at GBP 200,000/QALY gained in Figure 3a. This represents those simulations from the PSA where Nuvaxovid is less costly and more effective, more costly and more effective with an ICER below GBP 20,000, or less costly and less effective than mRNA vaccinations, where the latter has an ICER above GBP 20,000/QALY. This proportion is 70%, which can be interpreted as the probability that Nuvaxovid is cost-effective. The result was insensitive to the choice of cost-effectiveness threshold, with Nuvaxovid versus mRNA vaccination having a 66% or greater probability of being cost effective across the cost-effectiveness thresholds tested (GBP 0–GBP 300,000), reaching a maximum of 72%. The acceptability curve remains relatively flat as the threshold is varied, because most simulations produce a dominant result (Figure 3b).

### 3.3. Scenario Analyses

Eight scenarios were investigated; the Nuvaxovid strategy remained dominant over the mRNA strategy (Table 5).

The scenario with onset of waning at 2 months for Nuvaxovid resulted in potential net cost savings of GBP 87,891,163 and a QALY gain of 12,755 compared with mRNA vaccination (Nuvaxovid total costs GBP 4,830,427,740 and −464,813 QALY loss; mRNA total costs GBP 4,918,318,902 and −477,568 QALY loss). These net cost savings and net health benefit gains resulted from lower hospitalisations, ICU admissions, long COVID management, and other associated COVID-19 events (Table 5).

Relative to the base case Nuvaxovid-only strategy, scenarios investigating Nuvaxovid market shares of 50%, 33.3% and 10% resulted in progressively lower cost savings and fewer QALY gains (Table 5).

To account for the frequent updates to the JCVI recommendations [4,74,75], scenario analyses were conducted for the population eligible for annual vaccination. Changing the population did not affect the overall conclusions of the analysis. In scenario analyses, Nuvaxovid remained dominant over mRNA vaccines for all tested populations, including for the population aged ≥75 years (JCVI recommendation for 2025–2026 [75]) (Table 5).

### 3.4. Deterministic Sensitivity Analyses

**Figure 4:**
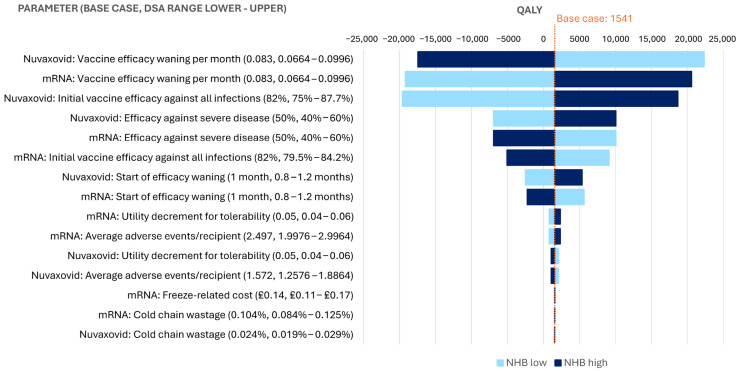
Net health benefit relative to base case. NHB = net health benefit; QALY = quality-adjusted life year.

ICERs generated in the DSA were the most sensitive to the vaccine efficacy waning rate and initial efficacy against all infections (Figure 4). Given the dominant ICER, the results of DSA are presented in terms of net health benefit (NHB) relative to base case of 1541 QALYs.

## 4. Discussion

This analysis examined the potential contribution of Nuvaxovid to the ongoing COVID-19–related public health challenge in the UK, demonstrating the value of its inclusion in an annual COVID-19 vaccination programme. In the base case, probabilistic sensitivity analysis showed cost savings of nearly GBP 1.4m and health gains of 1558 QALYs with Nuvaxovid only, compared with an mRNA-only vaccine strategy. The advantages of Nuvaxovid relative to mRNA vaccines in health terms primarily relate to its potential improved tolerability, while elimination of freeze-related operational costs and wastage provides cost savings. The potential improvements in tolerability of Nuvaxovid relative to mRNA vaccines may mean that, besides other regulatory approved populations, Nuvaxovid provides an additional vaccine choice for individuals who have previously experienced adverse events/reactogenicity with mRNA vaccines.

In the PSA, despite uncertainty around several of the parameters, Nuvaxovid was estimated to have an approximately 70% chance of resulting in a net population health gain. In the base case, we assumed no difference in the onset of efficacy waning between Nuvaxovid and mRNA vaccines, with onset of waning at 1 month. However, trends reported in some studies indicated that onset of waning could start later than 1 month for Nuvaxovid [12,42,43,44,45,72,73]. Scenario analysis with start of waning at 2 months for Nuvaxovid indicated potential cost savings of nearly GBP 88 million, driven by the potential reduction in hospitalisations and other healthcare resource use. Additionally, this scenario could potentially generate a gain of 12,755 QALYs. To account for the frequent updates to the JCVI-recommended population eligible for vaccination [4,74,75], scenarios varying the vaccine-eligible population were conducted. Changing the population did not affect the overall conclusions of the analysis. Nuvaxovid remained dominant over mRNA vaccines, including for the population aged ≥75 years.

Our model used a dynamic transmission approach to incorporate the indirect effects of vaccination on SARS-CoV-2 infection and transmission rates, as well as progression to symptomatic COVID-19 and COVID-19–related death, in line with existing guidelines for infectious disease modelling [23,24]. To our knowledge, this is the first dynamic transmission model to estimate the impact of vaccine selection on the vaccination strategy in the UK from an NHS perspective.

Economic models assessing the cost-effectiveness of protein-based vaccinations relative to no vaccination or mRNA vaccination have typically used a Markov state transition approach with static epidemiological inputs [20,21,22]. Population-based dynamic transmission models from France showed the potential impact of physical interventions such as facemask use from epidemiological and HCRU perspectives in the context of exiting from the pandemic and associated lockdowns [76,77]. Davies et al. (2020) [28] used dynamic transmission modelling to estimate rates of COVID-19 transmission, symptomatic infection rates and disease severity across age groups, based on international data, while a 2021 article by Sandmann et al. used a dynamic transmission approach to model the potential economic impact of introducing COVID-19 vaccination programmes in the UK [27]. Our model adapted the approaches from Davies et al. and Sandmann et al. to include a variety of vaccine profiles and vaccination strategies and address policy questions relevant for UK decision makers. Following calibration, the model achieved a good approximation to the reported UK incidence in 2022, as well as 2022 mortality rates and 2023/2024 hospitalisation rates, indicating external validity.

The population-based compartmental dynamic transmission model approach is the methodology of choice when the interventions being compared have a differential impact on transmission [24]. It was necessary to use COVID-19 epidemiological data from varying time periods to parameterise and calibrate the model. It would not have been possible to use such data with a static model that is insensitive to changes in the underlying COVID-19 epidemiology. Furthermore, several vaccine-related estimates such as equal vaccine efficacy, tolerability, and operational cost difference between Nuvaxovid and mRNA vaccines are uncertain. Within any one run of the PSA, the applied estimates are sampled independently and are therefore not identical, and the resulting difference in dynamics should account for any indirect effects, which cannot be achieved with a static model. Projecting the impact of a new vaccine on COVID-19 is challenging due to the evolving dynamics of COVID-19, including changes in immunity from prior infection and/or vaccination among the general population and changes in the circulating SARS-CoV-2 variants [61]. Additionally, changes in public health surveillance due to the reduction in testing and reporting since early 2023, when the pandemic status was lifted, contribute to the complexity.

To avoid underestimating incidence and mortality, we calibrated the model to the total number of incident cases recorded in 2022, collected during frequent testing in the general population and providing a more reliable estimate of overall COVID-19 epidemiology. However, using 2022 data may have overestimated burden of the disease for incidence and mortality. Hospitalisation rates were calibrated against 2023–2024 data because within-hospital COVID-19 testing has continued, and these more contemporary data are more likely to reflect any reductions in hospitalisation rates in recent years due to immunity from prior infection and/or vaccination, as well as circulation of potentially less virulent SARS-CoV-2 strains [78]. While we achieved close approximation to the reported data following model calibration, there were challenges in reproducing some age distributions, which likely had some impact on the outcomes.

To estimate vaccine efficacy, waning and tolerability, a combination of clinical trial and real-world data was used. This was because finding comparable sources for the tested vaccines proved to be challenging. We strived to address any potential uncertainties in our analyses, including using PSA for our base case and scenario analyses. The vaccine efficacy data used for Nuvaxovid and mRNA vaccines were based on phase 3 randomised, placebo-controlled trials undertaken in 2020 and 2021, conducted among vaccine-naïve individuals [15,38]. These clinical trial data were chosen over more recent real-world evidence as they provided estimates of efficacy against asymptomatic, as well as symptomatic, infections supported by polymerase chain reaction (PCR) and serology testing. It proved to be challenging to find comparable studies in the absence of a head-to-head trial. Indirect treatment comparisons of vaccines are problematic unless the data come from the same population at the same time. This observation is also a further limitation on our ability to estimate the future individual vaccine efficacies to apply in the model, for both Nuvaxovid and mRNA in the UK population.

Vaccine efficacy and waning may vary today and may not be the same for new SARS-CoV-2 variants, and may relate to factors such as antibody escape, particularly in the setting of recently emerged variants such as Omicron sublineages [9]. To remain effective, it is important for the vaccines to be adapted to the specific variant. Nuvaxovid adapted-vaccine to the XBB1.5 variant has achieved antibody titres superior to those reported for the original COVID-19 vaccines and SARS-CoV-2 strains [40,79], potentially supporting the level of efficacy applied [12]. Further, equivalent efficacy was assumed between mRNA vaccines and Nuvaxovid in the model; therefore, any differences would not impact the outcomes of the analysis. We tested the potential uncertainties in the sensitivity analyses.

Tolerability for Nuvaxovid and mRNA vaccines was based on a meta-analysis of reactogenicity events in clinical trials [39] and was a key driver of QALY gains with a Nuvaxovid-only versus mRNA-only vaccination strategy. However, head-to-head clinical trials have not been conducted to date. Storage of Nuvaxovid at standard refrigerator temperatures is another differentiator between Nuvaxovid and mRNA vaccines. Uncertainties around the vaccine effectiveness, tolerability, and freeze-related cost estimates were explored in the sensitivity analyses.

The timing of vaccination in the model is a further consideration. The UK vaccination programme recommends that COVID-19 vaccine should usually be offered no earlier than around 6 months after the last vaccine dose, but can be performed earlier (e.g., for populations at very high risk). Because of the potential variability in the timing of the vaccine administration among different populations, we opted to model an annual vaccination in the autumn when vaccination gives a higher return through reducing winter pressures.

Given the lack of clear seasonality of COVID-19, our assumptions may not accurately reflect the true effect and epidemiology. These uncertainties should be considered and caution exercised when interpreting the model findings for 2025 and beyond.

## 5. Conclusions

Our study’s findings indicate that a vaccine strategy incorporating Nuvaxovid into the existing COVID-19 vaccination programme in the UK may yield advantages over an mRNA-only vaccine strategy, driven mainly by the potential improved tolerability and operational efficiency of Nuvaxovid. Vaccination with Nuvaxovid emerged as the dominant strategy compared with exclusive reliance on mRNA vaccines. With this approach, fewer QALYs were lost, and costs related to freeze requirements were avoided. In probabilistic sensitivity analyses over 10,000 iterations, there was an approximately 70% probability of the Nuvaxovid vaccination strategy proving favourable compared with an mRNA-only strategy, regardless of the cost-effectiveness threshold.

## Figures and Tables

**Table 1:** Base case inputs for the dynamic transmission model and calibration.

Parameter	Base Case Value	
UK population [32]	Population size for England in 2021, extrapolated to the full UK population	
UK population with a pre-condition [33]	By age: 7–79%	
UK contact patterns [25,26]	Great Britain Close Contact matrix from the POLYMOD study using socialmixr 0.4.0 R package	
Incidence of SARS-CoV-2 infection [31]	UKHSA 2022 figures	
COVID-19–related mortality [31]	ONS 2022 mortality figures	
COVID-19–related hospitalisations/ICU admissions [3]	UKHSA 2023–2024 figures	
**Vaccine Uptake by Age Group**	No pre-condition	Pre-condition
12–18 years [31]	0.0%	5.34%
19–64 years [30]	0.0%	30.2%
65–69 years [30]	61.2%	61.2%
70–74 years [29]	70.1%	70.1%
75–79 years [29]	75.5%	75.5%
80–99 years [29]	75.7%	75.7%

ICU = intensive care unit; ONS = Office for National Statistics; UKHSA = United Kingdom Health Surveillance Agency.

**Table 4:** Base case results.

	Nuvaxovid	mRNA Vaccines	Incremental
**Probabilistic**			
Total Costs	GBP 4,979,904,295	GBP 4,981,292,618	−GBP 1,338,323
QALY losses	−483,644	−485,201	1558
ICER			Dominant

ICER = incremental cost-effectiveness ratio; QALY = quality-adjusted life year.

**Table 5:** Probabilistic scenario analysis results.

Base Case/ Scenario	Scenario Description	Incremental Costs	Incremental QALYs	ICER
Base case	Vaccination of those aged ≥65 years and 12–64 years with pre-conditions	−GBP 1,388,323	1558	Dominant
**Variation in waning**				
1	Onset of waning at 2 months for Nuvaxovid	−GBP 87,891,163	12,755	Dominant
**Variation in market share**				
2a	50% market share for Nuvaxovid	−GBP 906,780	686	Dominant
2b	33.3% market share for Nuvaxovid	−GBP 512,851	491	Dominant
2c	10% market share for Nuvaxovid	−GBP 109,666	115	Dominant
**Variation in vaccinated population**				
3a	Vaccination of those aged ≥65 years	−GBP 2,123,733	1070	Dominant
3b	Vaccination of those aged ≥75 years	−GBP 1,226,242	645	Dominant
3c	Vaccination of those aged 65–74 years	−GBP 639,379	603	Dominant
3d	Vaccination of those aged 12–64 years with pre-conditions	−GBP 540,473	331	Dominant

ICER = incremental cost-effectiveness ratio; QALY = quality-adjusted life year.

## Data Availability

All data relevant to this study were obtained from the published literature or other publicly available sources and have been presented in this article and Supplementary Materials.

## Supplementary Materials

The following supporting information can be downloaded at: https://www.mdpi.com/article/10.3390/vaccines13020187/s1, Figure S1: Relative contact frequency from the POLYMOD close contact matrix; Table S1: Reported COVID-19 cases (2022), COVID-19 hospitalisations (April 2023–April 2024) and deaths (2022) in England compared with model estimates; Table S2: Derivation of average tolerability/reactogenicity events from a meta-analysis of clinical trials; Figure S2: Ordinary differential equations included in the dynamic transmission model component; Figure S3: Changes in vaccine efficacy against overall infection in Nuvaxovid and mRNA vaccines over a 1-year period; Table S3: Parameter ranges used in the deterministic sensitivity analyses; Table S4: Parameter ranges used in the probabilistic analyses.
