# Supplementary material for: Cost-Effectiveness of Introducing Nuvaxovid to COVID-19 Vaccination in the United Kingdom: A Dynamic Transmission Model
Source: Vaccines (Basel). 2025 Feb 14;13(2):187. doi: 10.3390/vaccines13020187 (PMC11861217; doi:10.3390/vaccines13020187)
Supplement: Supplementary file 1 [file vaccines-13-00187-s001.zip › vaccines-3363859-supplementary.pdf]

Supplement

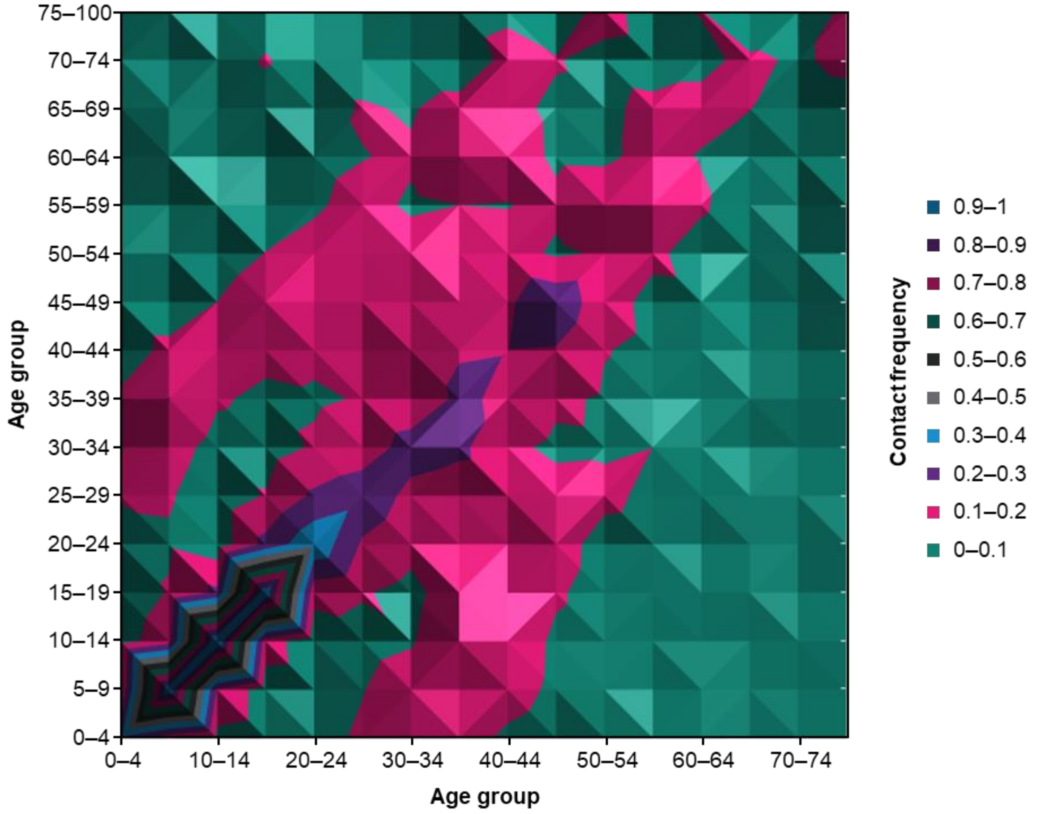

Figure S1: Relative contact frequency from the POLYMOD close contact matrix

Table S1: Reported COVID-19 cases (2022), COVID-19 hospitalisations (April 2023–April 2024) and deaths (2022) in England compared with model estimates.

Age group	COVID-19 Cases		Hospitalisations		Deaths	
	Reported COVID-19 cases (2022) [25]	Model results adjusted to England population	Reported hospitalisation (2023–2024) [3]	Model results Adjusted to England population	Reported COVID-19 deaths (2022) [71]	Model results adjusted to England population
0–4	200,794	176,014	—	151.50	19	2.97
5–9	499,332	429,835	—	393.38	11	7.26
10–14	562,263	524,250	—	147.77	8	14.34
15–19	433,383	441,901	—	53.71	21	14.33
20–24	517,183	553,101	—	116.12	34	34.24
25–29	652,544	702,002	—	760.77	44	50.20
30–34	749,718	799,782	—	1,195.50	81	143.42
35–39	748,736	788,735	—	1,680.05	135	153.85
40–44	705,145	746,935	—	2,567.95	182	258.37
45–49	629,265	668,510	—	3,415.75	319	279.42
50–54	640,809	712,212	—	4,678.77	547	1,055.14
55–59	588,733	652,057	—	6,953.29	770	1,177.38
60–64	477,016	519,111	—	12,789.16	1,211	2,662.21
65–69	345,700	274,900	—	7,126.05	1,756	1,568.58
70–74	301,107	220,226	—	12,219.57	2,904	3,024.82
75–79	231,028	169,481	—	17,448.09	4,375	2,629.51
80–84	151,794	110,973	—	11,820.80	5,638	8,092.94
85–89	116,885	86,353	—	9,557.79	6,979	6,720.85
≥90	100,275	75,285.26	—	8,218.26	8,460	5,603.70
**Total cases**	**8,651,710**	**8,651,662**	**118,141**	**101,294**	**33,494**	**33,494**

Figure S2. Ordinary differential equations defining the dynamic transmission model.

$$\frac{dS_{a,m}^{0}(t)}{dt} = -\lambda(t,a,0)S_{a,m}^{0}(t) + \rho_0 R_{a,m}^{0}(t) - v(a,t)S_{a,0}^{0}(\,t) + \rho_v S_{a,0}^{v}(\,t) \tag{1}$$

$$\frac{dE_{a,m}^{0}(t)}{dt} = \lambda(a,t,0)S_{a,m}^{0}(t) - \sigma E_{a,m}^{0}(t) - v(a,t)E_{a,0}^{0}(\,t) + \rho_v E_{a,0}^{v}(\,t) \tag{2}$$

$$\frac{dA_{a,m}^{0}(t)}{dt} = \psi(a)\sigma E_{a,m}^{0}(t) - \gamma_A A_{a,m}^{0}(t) - v(a,t)A_{a,0}^{0}(\,t) + \rho_v A_{a,0}^{v}(\,t) \tag{3}$$

$$\frac{dP_{a,m}^{0}(t)}{dt} = (1-\psi(a))\sigma E_{a,m}^{0}(t) - \eta P_{a,m}^{0}(t) - v(a,t)P_{a,0}^{0}(\,t) + \rho_v P_{a,0}^{v}(\,t) \tag{4}$$

$$\frac{dI_{a,m}^{0}(t)}{dt} = \eta P_{a,m}^{0}(t) - \gamma_I I_{a,m}^{0}(t) - \delta(a,m)I_{a,m}^{0}(t) - v(a,t)I_{a,0}^{0}(\,t) + \rho_v I_{a,0}^{v}(\,t) \tag{5}$$

$$\frac{dR_{a,m}^{0}(t)}{dt} = \gamma_I I_{a,m}^{0}(t) + \gamma_A A_{a,m}^{0}(t) - \rho_0 R_{a,m}^{0}(t) - v(a,t)R_{a,0}^{0}(\,t) + \rho_v R_{a,0}^{v}(\,t) \tag{6}$$

$$\frac{dS_{a,m}^{v}(t)}{dt} = -\lambda(t,a,v)S_{a,m}^{v}(t) + \rho_0 R_{a,m}^{v}(t) + v(a,t)S_{a,0}^{0}(\,t) - \rho_v S_{a,0}^{v}(\,t) \tag{7}$$

$$\frac{dE_{a,m}^{v}(t)}{dt} = \lambda(a,t,v)S_{a,m}^{v}(t) - \sigma E_{a,m}^{v}(t) + v(a,t)E_{a,0}^{0}(\,t) - \rho_v E_{a,0}^{v}(\,t) \tag{8}$$

$$\frac{dA_{a,m}^{v}(t)}{dt} = \psi(a)\sigma E_{a,m}^{v}(t) - \gamma_A A_{a,m}^{v}(t) + v(a,t)A_{a,0}^{0}(\,t) - \rho_v A_{a,0}^{v}(\,t) \tag{9}$$

$$\frac{dP_{a,m}^{v}(t)}{dt} = (1-\psi(a))\sigma E_{a,m}^{v}(t) - \eta P_{a,m}^{v}(t) + v(a,t)P_{a,0}^{0}(\,t) - \rho_v P_{a,0}^{v}(\,t) \tag{10}$$

$$\frac{dI_{a,m}^{v}(t)}{dt} = \eta P_{a,m}^{v}(t) - \gamma_I I_{a,m}^{v}(t) - \delta(a,m)I_{a,m}^{v}(t) + v(a,t)I_{a,0}^{0}(\,t) - \rho_v I_{a,0}^{v} \tag{11}$$

$$\frac{dR_{a,m}^{v}(t)}{dt} = \gamma_I I_{a,m}^{v}(t) + \gamma_A A_{a,m}^{v}(t) - \rho_0 R_{a,m}^{v}(t) + v(a,t)R_{a,0}^{0}(\,t) - \rho_v R_{a,0}^{v} \tag{12}$$

$m \in \{0 = \text{no preconditions}, 1 = \text{ with preconditions}\}$

$a \in \{single\ age, 0-99\}$

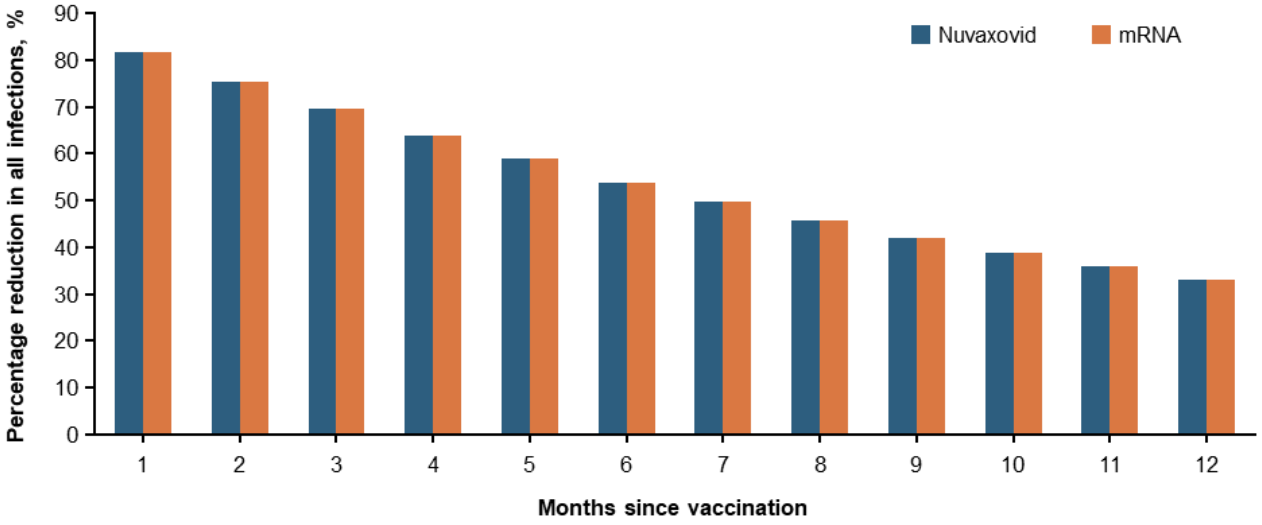

Figure S3. Changes in vaccine efficacy against overall infection in Nuvaxovid and mRNA vaccines over a 1-year period.

Table S2: Derivation of average tolerability/reactogenicity events from a meta-analysis of clinical trials

Reactogenicity event [32]	mRNA vaccine		Protein-based vaccine		Frequency of AE	
	No. of events	Sample size	No. of events	Sample size	mRNA	Protein-based
Fever	3425	21,143	1070	22,544	0.162	0.047
Fatigue	13,883	21,143	9936	22,545	0.657	0.441
Headache	12,874	21,143	8725	22,545	0.609	0.387
Pain	18,169	21,143	12,950	22,545	0.859	0.574
Redness	1914	21,143	1358	22,545	0.091	0.060
Swelling	2524	21,143	1404	22,545	0.119	0.062
Average adverse events / vaccine					2.497	1.572

AE, adverse event

Table S3: Parameters used in the deterministic sensitivity analyses.

			DSA Ranges	
	**Units**	**Base case**	**Lower**	**Upper**
**Disease state transition parameters**				
Contact rate*	%	By age: 0.000000135028 to 0.000005497611	−20%	+20%
Latent period*	days	4	3.2	4.8
Proportion of asymptomatic cases of all infections 0–9 yrs†	%	33%	23.7%	43.5%
Proportion of asymptomatic cases of all infections 10–19 yrs†	%	36%	26%	45.9%
Proportion of asymptomatic cases of all infections 20–29 yrs†	%	30%	21.3%	40.1%
Proportion of asymptomatic cases of all infections 30–49 yrs†	%	25%	16.6%	33.4%
Proportion of asymptomatic cases of all infections 50–64 yrs†	%	21%	15.6%	31%
Proportion of asymptomatic cases of all infections ≥65 yrs†	%	12%	3.8%	25.2%
Duration of presymptomatic stage*	days	1.5	1.2	1.8
Duration of asymptomatic stage*	days	5	4	6
Duration of symptomatic stage*	days	3.5	2.8	4.2
Time to death*	days	22	17.6	26.4
Waning immunity from infection ‡	days	315	84	364
COVID-19 mortality*	%	By age: 0.0003790 to 0.336945	−20%	+20%
**Vaccine efficacy: Nuvaxovid**				
Nuvaxovid: Initial efficacy against all infections†,§	%	82%	75.0%	87.7%
Nuvaxovid: Efficacy against severe disease*	%	50%	40%	60%
Nuvaxovid: Start of efficacy waning*	months	1	0.8	1.2
Nuvaxovid: Vaccine efficacy waning per month*	rate	0.083	0.0664	0.0996
**Vaccine efficacy: mRNA**				
mRNA: Initial efficacy against all infections †	%	82%	79.5%	84.2%
mRNA: Efficacy against severe disease*	%	50%	40%	60%
mRNA: Start of efficacy waning*	months	1	0.8	1.2
mRNA: Vaccine efficacy waning per month*	rate	0.083	0.0664	0.0996
**Utility decrements**				
Symptomatic case*	yrs	0.008	0.0064	0.0096
Non-fatal hospitalisation*	yrs	0.0201	0.01608	0.02412
Non-fatal ICU*	yrs	0.15	0.12	0.18
Long COVID*	yrs	0.13	0.104	0.156
Nuvaxovid tolerability *	utility decrement	0.05	0.04	0.06
mRNA tolerability *	utility decrement	0.05	0.04	0.06

	Units	Base case	DSA Ranges	
			Lower	Upper
Nuvaxovid: Average adverse events / recipient*	AEs/vaccination	1.572	1.2576	1.8864
mRNA: Average adverse events / recipient*	AEs/vaccination	2.497	1.9976	2.9964
**Healthcare resource use and costs**				
Hospitalisations*	%	By age: 0.03% to 11.89%	−20%	+20%
ICU admissions*	%	By age: 0.58% to 7.88%	−20%	+20%
Long COVID*	%	10%	−20%	+20%
General practitioner visits*	%	15.5%	12.4%	18.6%
Emergency visits*	%	2.7%	2.2%	3.2%
Cost of hospitalisation*	£	3,534	2,827	4,240
Cost of ICU*	£	24,494	19,595	29,393
mRNA: cold chain wastage	%	0.104	0.084	0.125
Nuvaxovid: cold chain wastage	%	0.024	0.019	0.029
mRNA: freeze-related costs	£	0.14	0.11	0.17

DSA = deterministic sensitivity analysis; ICU = intensive care unit; QALY = quality-adjusted life year.
*varied by +/− 20%; † 95% CI; ‡the upper bound of the range has been limited to the timeframe of the model (e.g., 1 year); §The 95% CI used was referenced from a 2021 article by Heath et al. [12]

Table S4: Parameters used in the probabilistic analyses.

		PSA Parameters*		
	**s.e.**	**Parameter 1**	**Parameter 2**	**Distribution**
**Disease state transition parameters**				
Contact rate†	0.1	0.0000	0.2000	Normal
Latent period†	2	4	1	Gamma
Proportion of asymptomatic cases of all infections 0–9 yrs‡	0.0495	29.45	59.79	Beta
Proportion of asymptomatic cases of all infections 10–19 yrs‡	0.0498	33.15	58.94	Beta
Proportion of asymptomatic cases of all infections 20–29 yrs‡	0.0470	28.22	65.85	Beta
Proportion of asymptomatic cases of all infections 30–49 yrs‡	0.0420	26.32	78.97	Beta
Proportion of asymptomatic cases of all infections 50–64 yrs‡	0.0385	23.29	87.63	Beta
Proportion of asymptomatic cases of all infections ≥65 yrs‡	0.0535	4.30	31.56	Beta
Duration of presymptomatic stage†	0.75	4	0.375	Gamma
Duration of asymptomatic stage†	2.50	4	1.250	Gamma
Duration of symptomatic stage†	1.75	4	0.875	Gamma
Time to death†	4.69	22.004	1.000	Gamma
Waning immunity from infection§	70	10	31.5	Gamma
COVID-19 mortality†	0.1	0.0000	0.1000	Normal
**Vaccine efficacy: Nuvaxovid**				
Nuvaxovid: Reduction in infections, %‡	0.03175	119.2440	26.1755	Beta
Nuvaxovid: Reduction in disease severity, %†	0.05	49.5000	49.5000	Beta
Nuvaxovid: Start of waning†	0.1	100.00	0.0100	Gamma
Nuvaxovid: Vaccine efficacy waning†		−2.712	−2.307	Lognormal
**Vaccine efficacy: mRNA**				
mRNA: Reduction in infections, %‡	0.01175	875.8264	192.2546	Beta
mRNA: Reduction in disease severity, %†	0.05	49.5000	49.5000	Beta
mRNA: Start of waning†	0.1	100.00	0.0100	Gamma
mRNA: Vaccine efficacy waning†		−2.712	−2.307	Lognormal
**Utility decrements**				
Symptomatic case†	–	99.192	12299.808	Beta
Non-fatal hospitalisation†	–	97.970	4776.154	Beta
Non-fatal ICU†	–	84.850	480.817	Beta
Long COVID†	–	86.870	581.361	Beta
Nuvaxovid tolerability†	–	94.950	1804.050	Beta
mRNA tolerability†	–	94.950	1804.050	Beta
Nuvaxovid: Average adverse events / recipient†	0.157	–	–	Normal
mRNA: Average adverse events / recipient†	0.250	–	–	Normal

		PSA Parameters*		
	**s.e.**	**Parameter 1**	**Parameter 2**	**Distribution**
**Healthcare resource use and costs**				
Hospitalisations†	–	1.00000	0.06700	Normal
ICU admissions†	–	1.00000	0.06700	Normal
Long COVID†	–	1.00000	0.06700	Normal
General practitioner visits†	–	85	463	Beta
Emergency visits†	–	15	533	Beta
Cost of hospitalisation †	–	7.947	8.352	Lognormal
Cost of ICU†	–	9.883	10.289	Lognormal
mRNA: cold chain wastage	0.0001044	99.89	95,585	Beta
Nuvaxovid: cold chain wastage	0.0000241	99.98	414,737	Beta
mRNA: freeze-related cost*	0.013908	100.00	0.0014	Gamma

ICU = intensive care unit; PSA = probabilistic sensitivity analysis; QALY = quality-adjusted life year; s.e. = standard error
*Standard errors are reported only for normal and truncated normal distribution; †Varied by +/− 20%; ‡95% CI; §The upper bound of the range has been limited to the timeframe of the model (e.g., 1 year)
